# Managing Bone Metastases with Denosumab: Real-World Data and Critical Monitoring Points in Breast, Lung, and Prostate Cancers

**DOI:** 10.3390/medicina61091637

**Published:** 2025-09-10

**Authors:** Sibel Oyucu Orhan, Bedrettin Orhan, Şeyma Esenbuğa, Seda Sali, Burcu Caner, Birol Ocak, Ahmet Bilgehan Şahin, Adem Deligönül, Türkkan Evrensel, Erdem Çubukçu

**Affiliations:** 1 Department of Medical Oncology, Bursa City Hospital, Bursa 16250, Turkey; seda_ist1987@hotmail.com; 2 Department of Hematology, Bursa Yuksek Ihtisas Training and Research Hospital, University of Health Science, Bursa 16000, Turkey; borhan18@gmail.com; 3 Department of Internal Medicine, Faculty of Medicine, Bursa Uludag University, Bursa 16059, Turkey; ss.esenbuga@gmail.com; 4 Department of Medical Oncology, Aydın Atatürk Public Hospital, Aydin 09020, Turkey; drburcucaner@gmail.com; 5 Department of Medical Oncology, Bursa Yuksek Ihtisas Training and Research Hospital, University of Health Science, Bursa 16000, Turkey; birol08ocak@gmail.com; 6 Department of Medical Oncology, Faculty of Medicine, Bursa Uludag University, Bursa 16059, Turkey; absahin@uludag.edu.tr (A.B.Ş.); ademdeligonul@yahoo.com (A.D.); turkkanevrensel@gmail.com (T.E.); erdemcubukcu@uludag.edu.tr (E.Ç.)

**Keywords:** denosumab, skeletal-related events, hypocalcemia, bone metastasis

## Abstract

*Background and Objectives*: Advanced solid organ tumors, particularly breast, lung, and prostate cancers, frequently metastasize to bone, leading to debilitating skeletal-related events (SREs). Denosumab, a RANKL inhibitor, is crucial in preventing SREs. This study aimed to comparatively evaluate the efficacy and adverse effect profiles of denosumab in patients with bone metastases originating from these three common cancer types. *Materials and Methods*: This retrospective study included 146 patients treated with denosumab for bone metastases. Data on demographics, SREs before and during denosumab treatment, serum creatinine, calcium, and magnesium levels (at baseline, 3, and 6 months), other adverse effects, and survival were analyzed. *Results*: Before denosumab, SREs were present in 36.3% of patients (breast: 43.4%, prostate: 28%, lung: 33.8%). During denosumab treatment, SRE rates markedly decreased across all groups (breast: 9.4%, prostate: 16.0%, lung: 8.8%), with no significant intergroup difference in on-treatment SREs. Significant decreases in serum calcium levels were observed at 3 and 6 months post-denosumab initiation in breast (*p* < 0.0001) and lung cancer patients (*p* = 0.001). Mean creatinine levels significantly decreased in lung (*p* < 0.0001) and prostate (*p* = 0.020) cancer patients at 3 and 6 months. Overall survival significantly differed, with lung cancer patients having the shortest median survival (*p* < 0.005). *Conclusions*: Denosumab effectively reduces the incidence of SREs in patients with bone metastases from breast, lung, and prostate cancer. However, clinicians must diligently monitor for hypocalcemia, a notable adverse effect, particularly at 3 and 6 months after starting denosumab, with specific caution warranted in patients with lung cancer.

## 1. Introduction

Bone metastases are prevalent in some patients with advanced cancer with an incidence of approximately 55%, 90%, and 33% in breast, prostate, and lung cancers, respectively [1,2,3]. Patients with bone metastases may experience irreversible skeletal complications known as SREs, including pathologic fractures, spinal cord compression, and/or radiation or surgical intervention to bone [4]. Over the last three decades, intravenous bisphosphonates, including pamidronate and zoledronic acid, were developed as effective treatments to delay or prevent SREs in patients with bone metastases as an important step in improving palliative cancer care. SREs are serious sequelae in patients with bone metastases (BMs) of solid organ tumors and are associated with reduced quality of life, increased pain, and decreased survival [4].

Denosumab is a monoclonal antibody with affinity for receptor activator of nuclear factor-kappa ligand (RANKL). Osteoblasts secrete RANKL and RANKL activates osteoclast precursors and subsequent osteolysis that induces release of bone-derived growth factors, including insulin-like growth factor-1 (IGF1) and transforming growth factor-beta (TGF-beta), and increases serum calcium levels. Denosumab binds to RANKL, blocks the interaction between RANKL and RANK (a receptor located on osteoclast surfaces), and prevents osteoclasts, leading to decreased bone resorption and increased bone mass in osteoporosis. RANKL inhibition decreases osteoclastic activity, leading to decreased SREs and tumor-induced bone destruction in solid tumors with bone metastases [5,6]. Key phase III studies reported that denosumab had better efficacy than zoledronic acid, a widely used bisphosphonate [4,5,7,8]. Denosumab was also superior to zoledronic acid in reducing the risk of multiple SREs by 18% (Hazard ratio [HR]: 0.82; 95% confidence interval [CI]: 0.75–0.89; *p* < 0.001). Furthermore, denosumab significantly delayed the time to first SREs or malignancy-related hypercalcemia (26.5 months for denosumab vs. 19.3 months for zoledronic acid; HR: 0.83; 95% CI: 0.76–0.90; *p* < 0.001) [4].

Phase III studies reported that the most prevalent adverse effects of denosumab included nausea, anemia, fatigue, back pain, and decreased appetite with the incidence similar to that of zoledronic acid [4]. Adverse effects including renal failure, hypocalcemia, and osteonecrosis of the jaw were also observed during denosumab treatment, and previous studies which compared zoledronic acid to denosumab reported that zoledronic acid was associated with higher prevalence of renal adverse effects in breast cancer study. The incidence of renal adverse events in patients with baseline renal clearance ≤ 60 mL/min was greater in the zoledronic acid group (20.0%) compared to the denosumab group (5.9%) [7]. An integrated analysis of phase III trials reported that the hypocalcemia prevalence was higher with denosumab than with zoledronic acid (9.6% vs. 5.0%) [6]. Another study reported that the cumulative incidence of confirmed osteonecrosis of the jaw was relatively low (89 patients out of a total of 5723 patients; 1.6%) and that there was no significant difference between denosumab and zoledronic acid treatments (1.8% vs. 1.3%, respectively; *p* = 0.13) [9].

Existing research on denosumab for treating BMs frequently encompasses diverse cancer types and often centers on comparisons with zoledronic acid. This approach, while informative, has limitations. Most studies on denosumab combine heterogeneous cancer types, limiting insights into tumor-specific efficacy and safety. Few reports directly compare its outcomes in breast, lung, and prostate cancers—the three tumors where bone metastases are most common. For example, a notable study investigating denosumab-induced hypocalcemia included patients with multiple myeloma and focused on a comparison with zoledronic acid [4], rather than concentrating solely on solid tumors. Furthermore, although a recent systematic review concentrates on bone-modifying agents for breast, lung, and prostate cancer, it does not provide an in-depth investigation of the adverse effect profile of denosumab [10].

Therefore, this study aimed to comparatively evaluate the efficacy and adverse effect profiles of denosumab in patients with BMs originating from breast, lung, and prostate cancer.

## 2. Materials and Methods

Patients with solid organ malignancies and BMs were retrospectively reviewed by the Medical Oncology Department of Bursa Uludag University Hospital between 2012 and 2022. Patients older than 18 years of age with a histologically confirmed diagnosis of breast, lung, or prostate cancer and radiologically confirmed bone metastases were included in the study. Additionally, patients with baseline chronic kidney disease were also included. Patients were excluded if they had any prior use of bisphosphonates, had a concurrent primary malignancy, had incomplete clinical data, or were lost to follow-up. Age, sex, ECOG performance status, comorbidities, diagnoses, chemotherapies, SREs before denosumab; creatinine, calcium, and magnesium levels at the time of diagnosis and at months 3 and 6; and adverse effects during denosumab treatment, palliative bone surgery or radiotherapy status, total follow-up time, and overall survival were evaluated. Denosumab treatment started immediately after detecting an SRE. Hypocalcemia and hypomagnesemia were defined and classified pursuant to the Common Terminology Criteria for Adverse Effects (CTCAE) version 5.0 of National Cancer Institute [11]. Renal impairment was defined as a serum creatinine level of ≥1.4 mg/dL or an increase in serum creatinine levels of >0.5 mg/dL from the baseline serum creatinine level of <1.4 mg/dL, or an increase in serum creatinine to twice or more the baseline value [12]. Radiological evaluation and confirmation of bone metastases were performed. Dental evaluation was assessed before denosumab initiation. Patients received subcutaneous denosumab 120 mg every 4 weeks. All patients in the study were advised to take ≥1000 mg calcium + 880 IU vitamin D daily.

The Statistical Package for the Social Sciences (SPSS, IBM Corp., Armonk, NY, USA) software for Windows Version 25.0 was used for the statistical analyses. Descriptive statistics are presented as number (n) and percentage (%) for categorical variables and mean ± standard deviation (SD) or median (range) for continuous variables. Shapiro–Wilk test was used to test the normal distribution hypothesis for the study data. Friedman test was used for repeated data that did not meet normal distribution hypothesis. Each group was compared pairwise, when an intergroup difference was detected as a result of the Friedman test. Because the data were dependent in pairwise comparisons, the Wilcoxon test was used. Because the data did not meet the normal distribution hypothesis, Kruskal–Wallis test was used between the cancer types. When differences were detected using the Kruskal–Wallis test, Mann–Whitney *U* or Dunn’s test was used in pairwise comparisons. Chi squared test or Fisher’s exact test was used to compare categorical variables. Kaplan–Meier method was used to compare survival and disease-free survival between various clinical factors. A *p*-value < 0.05 was considered statistically significant.

## 3. Results

A total of 146 patients with breast, lung, or prostate cancer with available data were included in the study. Demographics and clinical characteristics of the patients are presented in Table 1. The mean age was 58.3 ± 13.5 years for the entire population and 50.2 ± 14.3, 67.6 ± 8.1, 61.3 ± 11.0 years for patients with breast, prostate, and lung cancers, respectively. Among the patients, 53 (36.3%) were diagnosed with breast cancer, 25 (17.1%) with prostate cancer, and 68 (46.6%) with lung cancer. Further, 22 patients had chronic kidney disease, 10 had chronic obstructive pulmonary disease, 33 had coronary artery disease, 67 had hypertension, and 31 had diabetes mellitus. After diagnosis, 66 patients received only chemotherapy, 17 patients received chemotherapy + targeted therapy, 15 patients received chemotherapy and immunotherapy, 1 patient received only hormonotherapy, and 47 patients received chemotherapy and hormonotherapy. Before denosumab treatment, 53 (36.3%) patients had SREs. The rates of SREs in breast, prostate, and lung cancers were 43.4% (23/53), 28% (7/25), and 33.8% (23/68), respectively (Table 2). At the onset of denosumab treatment and at months 3 and 6, median serum calcium levels were 9.3, 9.0, and 9.0, respectively, serum magnesium levels were 2.0, 2.0, and 2.0, respectively, and serum creatinine levels were 0.78, 0.68, and 0.64, respectively. Because of SREs, 89 (61%) patients received radiotherapy and 13 (9%) patients underwent surgical operations. Median duration of denosumab use was 12.3 (0.2–61.8) months. The median total follow-up period was 27.5 (1.5–172.2) months. None of the patients had ever used bisphosphonates.

**Figure 1:**
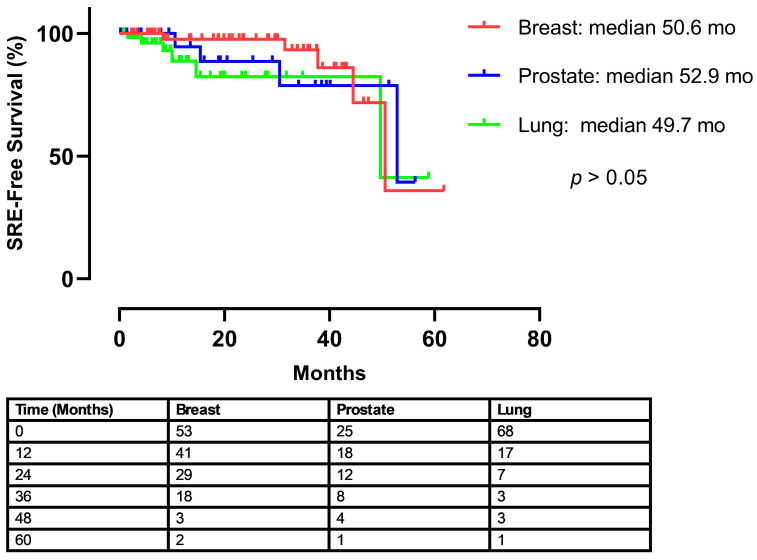
Kaplan–Meier analysis of time to first Skeletal-Related Event (SRE) after the start of Denosumab therapy and number of patients at risk by time.

According to our analysis of denosumab adverse effects by cancer types, hypocalcemia was observed in 5.6% (3/53) of patients with breast cancer, 4% (1/25) of those with prostate cancer, and 10.2% (7/68) of those with lung cancer. There was no significant intergroup difference (*p* = 0.482, x^2^ = 1.459) (Table 2). Creatinine increase in patients with breast, prostate, and lung cancer was 5.7% (3/53), 16% (4/25), and 19.1% (13/68), respectively, and there was no significant intergroup difference (*p* = 0.101, x^2^ = 4.698). Although there was no significant intergroup difference by infections during denosumab treatment (*p* = 0.593, x^2^ = 1.308), the highest rate (20%) was observed in patients diagnosed with prostate cancer. Further, although there was no significant intergroup difference among patients by occurrence of malignant hypercalcemia (*p* = 0.173, x^2^ = 3.533), the incidence was highest in patients with lung cancer (19.1%, 13/68). Adverse effects, categorized by grade, are presented in Table 3. The rate of SREs in patients with breast cancer, prostate cancer, and lung cancer during denosumab treatment was 9.4% (5/53), 16.0% (4/25), and 8.8% (6/68), respectively, and there was no significant intergroup difference by SREs during denosumab treatment (*p* = 0.668, x^2^ = 1.236) (Table 2). To evaluate the timing of skeletal-related events (SREs) following the initiation of treatment, a time-to-event analysis was performed. The Kaplan–Meier curve illustrates the SRE-free survival for each of the three cancer cohorts (Figure 1). The median time to the first on-treatment SRE was 50.6 months for patients with breast cancer, 52.9 months for prostate cancer, and 49.7 months for lung cancer. A log-rank test revealed no statistically significant difference in the time to first SRE among the groups (*p* > 0.05).

**Figure 2:**
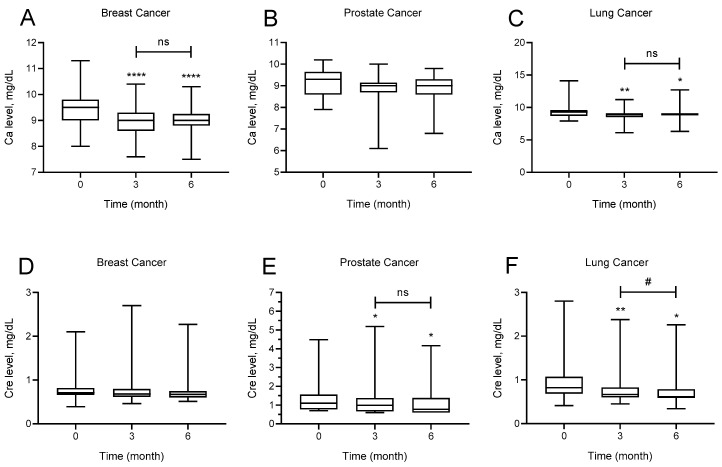
Effect of Denosumab administration on calcium (Ca) and creatinine (Cre) levels in different cancer types (breast, prostate, and lung). (**A**): Changes in calcium levels in breast cancer patients at baseline (0), 3 months, and 6 months. (**B**): Changes in calcium levels in prostate cancer patients at baseline (0), 3 months, and 6 months. (**C**): Changes in calcium levels in lung cancer patients at baseline (0), 3 months, and 6 months. (**D**): Changes in creatinine levels in breast cancer patients at baseline (0), 3 months, and 6 months. (**E**): Changes in creatinine levels in prostate cancer patients at baseline (0), 3 months, and 6 months. (**F**): Changes in creatinine levels in lung cancer patients at baseline (0), 3 months, and 6 months. Data were analyzed with the Friedman test for multiple differences in times. Wilcoxon test was used for pairwise comparisons of times. * *p* < 0.05, ** *p* < 0.01, **** *p* < 0.0001 as compared to initial time for Denosumab administration; # *p* < 0.05 as compared to 6th month groups, ns: not significant.

The effect of denosumab administration on calcium and creatinine levels at baseline and at months 3 and 6 for each cancer type is shown in Figure 2. Calcium levels of patients with breast cancer at months 3 and 6 after denosumab use were significantly lower than baseline calcium levels (*p* < 0.0001). Mean baseline calcium levels were 9.4 ± 0.63 mg/dL, whereas mean calcium levels at months 3 and 6 were 8.97 ± 0.53 and 9.01 ± 0.52 mg/dL, respectively. There was no significant difference in calcium levels of patients with prostate cancer at months 3 and 6 after denosumab use (*p* = 0.062). Calcium levels of patients with lung cancer at months 3 and 6 after denosumab use were significantly lower than baseline calcium levels (*p* = 0.001). Mean baseline calcium levels were 9.3 ± 0.96 mg/dL, whereas calcium levels at months 3 and 6 were 8.84 ± 0.73 and 9.02 ± 0.71, respectively.

There was no significant difference by creatinine levels of patients with breast cancer after denosumab use at months 3 and 6 (*p* = 0.071). Creatinine levels of patients with prostate cancer at months 3 and 6 after denosumab use were significantly lower than baseline creatinine levels (*p* = 0.020). Mean baseline creatinine levels were 1.35 ± 0.83 mg/dL, whereas mean creatinine levels at months 3 and 6 levels were 1.22 ± 0.96 mg/dL and 1.16 ± 0.89 mg/dL, respectively. Creatinine levels of patients with lung cancer at months 3 and 6 after denosumab use was significantly lower than baseline creatinine levels, and the creatinine level at month 6 was also significantly lower than that of month 3 (*p* < 0.0001). Mean baseline creatinine levels were 0.95 ± 0.43, whereas mean creatinine levels at months 3 and 6 were 0.82 ± 0.40 mg/dL and 0.75 ± 0.33 mg/dL, respectively.

**Figure 3:**
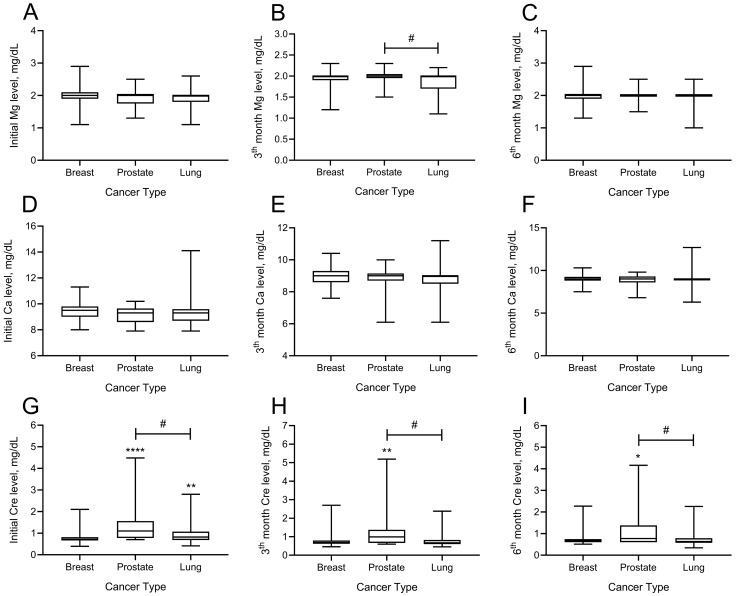
Effect of Denosumab administration on magnesium (Mg), calcium (Ca) and creatinine (Cre) levels in different cancer types (breast, prostate, and lung). (**A**): Initial (baseline) serum magnesium levels for patients with breast, prostate, and lung cancer. (**B**): Comparison of serum magnesium levels at the 3-months follow-up in patients with breast, prostate, and lung cancer. (**C**): Comparison of serum magnesium levels at the 6-months follow-up in patients with breast, prostate, and lung cancer. (**D**): Initial (baseline) serum calcium levels in mg/dL among patients with breast, prostate, and lung cancer. (**E**): Comparison of serum calcium levels at the 3-months follow-up in patients with breast, prostate, and lung cancer. (**F**): Comparison of serum calcium levels at the 6-months follow-up in patients with breast, prostate, and lung cancer. (**G**): Initial (baseline) serum creatinine levels across breast, prostate and lung cancer groups. (**H**): Comparison of serum creatinine levels at the 3-months follow-up in patients with breast, prostate, and lung cancer. (**I**): Comparison of serum creatinine levels at the 6-months follow-up in patients with breast, prostate, and lung cancer. Data were analyzed with the Kruskal–Wallis test for multiple differences in cancer type. Mann–Whitney U test was used for pairwise comparisons of cancer types. * *p* < 0.05, ** *p* < 0.01, **** *p* < 0.0001 as compared to Breast Cancer; # *p* < 0.05 as compared to Prostate Cancer.

The effects of denosumab administration on magnesium, calcium, and creatinine at months 3 and 6 are shown in Figure 3. There was no difference between magnesium levels among patients with breast, prostate, and lung cancer at the onset of denosumab treatment (*p* > 0.05). Analysis of magnesium levels at month 3 showed that the difference between patients with prostate and lung cancers was significant (*p* = 0.032). Patients with lung cancer had lower magnesium levels at month 3 than patients with prostate cancer (mean ± SD; 1.98 ± 0.15 vs. 1.84 ± 0.25 mg/dL). There was no significant intergroup difference by magnesium levels at month 6.

Regarding the calcium levels, there was no significant difference between the cancer groups at baseline and months 3 or 6. Analysis of the creatinine levels showed a significant intergroup difference by baseline creatinine levels. Baseline creatinine levels of patients with both prostate and lung cancer were significantly higher than those of patients with breast cancer (mean ± SD; prostate 1.35 ± 0.83, lung cancer 0.95 ± 0.43, breast 0.79 ± 0.29; *p* < 0.05). Analysis of creatinine levels at month 3 showed that the creatinine levels of patients diagnosed with prostate cancer were significantly higher than those of both patients with breast and lung cancer (mean ± SD; prostate 1.22 ± 0.96, lung cancer 0.98 ± 0.40, breast 0.77 ± 0.34, prostate vs. breast cancer *p* = 0.010, prostate vs. lung cancer *p* = 0.009). Analysis of creatinine levels at month 6 showed that patients with prostate cancer had significantly higher creatinine levels than patients with breast and lung diagnoses (mean ± SD; prostate 1.16 ± 0.89, breast 0.74 ± 0.27, lung 0.75 ± 0.33, prostate vs. breast cancer *p* = 0.017, prostate vs. lung cancer *p* = 0.002) (Figure 2).

Patient groups were also analyzed for overall survival (OS) and post-denosumab survival. The median OS for breast cancer, prostate cancer, and lung cancer was 112.9 (range: 85.3–124) months, 63.4 (range: 44.7–70.1) months, and 12.9 (range: 15.4–29.3) months, respectively, and the intergroup difference was statistically significant (*p* < 0.005). The median survival after denosumab use in breast, prostate, and lung cancer was 42.4 (range: 31.7–44.9) months, 20.5 (range: 19.2–35.6) months, and 6.3 (range: 7.5–14.0) months, respectively, and the intergroup difference was statistically significant (*p* < 0.005).

## 4. Discussion

The significant burden and effect of BMs and SREs on quality of life in patients with breast, prostate, and lung cancer require effective therapeutic management. Agents that target bone, delay complications, relieve symptoms, and improve quality of life, and these agents should be started at the time of diagnosis of metastatic bone disease [13]. This retrospective study provides real-world insights into the efficacy and safety profile of denosumab across these three common solid tumors with frequent bone involvement within a Turkish patient cohort. The most notable observation was the differential incidence of hypocalcemia across tumor types. Despite comparable baseline renal function and calcium supplementation (≥1000 mg calcium + 880 IU vitamin D daily), hypocalcemia occurred most frequently in lung cancer patients (10.2% vs. 5.6% in breast and 4% in prostate cancer). This contrasts with prior studies that identified prostate cancer as a high-risk group for hypocalcemia due to its osteoblastic microenvironment [4,14]. Our findings suggest that lung cancer patients’ shorter median post-denosumab survival (6.3 months) may limit their ability to maintain adequate calcium levels, potentially due to disease progression or treatment discontinuation. This hypothesis aligns with Nakamura et al.’s recent work linking shorter survival to poorer adherence to supplementation protocols [15].

Male predominance was generally observed in studies examining the use of denosumab in metastatic solid organ tumors [4,5]. In the present study, 76 (52.1%) out of 146 patients were male. The mean age was 58.3 (±13.5) years and both age and male predominance were consistent with those reported in the literature [16,17].

A study by Mizuta et al. on lung, breast, prostate, renal, and colorectal cancers reported the median follow-up period as 13 months and the rate of hypocalcemia as 6.9% for all solid organ tumors, and the rate of SREs during denosumab treatment was only 0.8% [16]. Henry et al. reported the rate of SREs before denosumab use as 47%, and there was a 15% decrease in SREs after denosumab use. In the same study, the most prevalent SREs during denosumab treatment were bone radiotherapy (14%) and pathologic fractures (12%) [18]. Another study reported that denosumab decreased the risk of SRE by 17%, the risk of multiple SREs by 18%, and the median time to first SRE above 8 months [19]. The prevention of SREs remains a cornerstone of palliative care for patients with BMs. Our data demonstrate a substantial decrease in SRE rates during denosumab treatment across all three cancer types (breast: 43.4% pre-treatment vs. 9.4% during treatment; prostate: 28.0% vs. 16.0%; lung: 33.8% vs. 8.8%). This reduction aligns with the established efficacy of denosumab observed in pivotal phase III trials, where it proved superior or non-inferior to zoledronic acid in delaying time to first SRE and reducing the risk of multiple SREs [4,5,7,20]. While our study lacks a direct comparator arm (e.g., zoledronic acid), the observed reduction in SREs from baseline provides clinically relevant evidence supporting denosumab’s role in routine practice, consistent with findings from other real-world observational studies [5,16,18]. Interestingly, despite differing baseline SRE rates potentially reflecting distinct tumor biology [13,14,21], the on-treatment SRE rates were numerically similar across the groups (8.8–16.0%, *p* = 0.668), suggesting a consistent effect of RANKL inhibition on suppressing osteoclast activity regardless of the primary tumor, although the underlying bone microenvironment interactions may differ [14]. The slightly higher rate in the prostate cancer group during treatment might warrant further investigation in larger cohorts, considering the mixed osteoblastic/osteoclastic nature of its metastases [8,14].

Hypocalcemia is a well-recognized adverse effect of denosumab, attributed to its potent inhibition of osteoclast-mediated calcium release from bone [6,22]. Our study observed clinical hypocalcemia (any grade) in 5.6% of breast, 4.0% of prostate, and 10.2% of lung cancer patients, with no statistically significant difference between groups (*p* = 0.482). Furthermore, we observed a statistically significant decrease in mean calcium levels from baseline at 3 and 6 months in both breast (*p* < 0.0001) and lung cancer patients (*p* = 0.001), reinforcing the need for proactive calcium and vitamin D supplementation and regular monitoring, as recommended by guidelines and previous studies [22,23]. This timeframe aligns with the known pharmacodynamics of denosumab, where maximal suppression of bone resorption occurs relatively quickly, while calcium incorporation into newly formed bone matrix might continue for months [22]. Contrary to some large cohort analyses that identified prostate cancer as an independent risk factor for hypocalcemia [22,24], our prostate cancer group did not show a significant decrease in mean calcium levels (*p* = 0.062) and had the lowest rate of clinical hypocalcemia (4.0%). This finding might be influenced by the smaller sample size of our prostate cancer cohort (n = 25). Furthermore, it is possible that unmeasured variables—including variations in baseline renal function, vitamin D status, extent of bone metastases, or adherence to supplementation protocols—may differ in our cohort compared to those in larger studies, thus explaining the discrepancy [22,24]. Nevertheless, the significant drop observed in breast and lung cancer groups at 3 and 6 months highlights a critical window for potential hypocalcemia development across different tumor types. The trend towards a higher rate of hypocalcemia in lung cancer patients is noteworthy and supported by data from a phase 3 study, which reported rates of 8.6% in the denosumab arm versus 3.8% in the zoledronic acid arm [25]. The most potent mechanism explaining this heightened vulnerability is likely ‘consumption hypocalcemia.’ This phenomenon is particularly relevant in patients with high osteoblastic activity, where denosumab’s powerful blockade of bone resorption cannot compensate for the persistent calcium demand from ongoing bone formation [26]. Given that extensive osteoblastic metastases are a known risk factor, the clinical severity of this mechanism is starkly illustrated by the case from Mori et al., which detailed severe hypocalcemia requiring four months of treatment in a lung cancer patient with diffuse bone metastases [27]. Furthermore, denosumab’s long half-life creates the potential for this hypocalcemia to be prolonged, compounding the clinical challenge. The higher numerical rate in lung cancer patients could potentially relate to factors common in this population, such as poorer nutritional status or higher baseline bone turnover, although our study could not definitively assess these. A recent multicenter study also highlighted renal function and baseline calcium levels as key risk factors, but did not find a direct link to cancer type itself [15].

Regarding renal safety, denosumab is generally considered to have a favorable profile compared to intravenous bisphosphonates, particularly zoledronic acid [5,7]. Our results showed a statistically significant decrease in mean creatinine levels at 3 and 6 months compared to baseline in the lung (*p* < 0.0001) and prostate cancer groups (*p* = 0.020), while numerically higher rates of creatinine increase (based on study definition/CTCAE criteria) were reported in these same groups (Lung: 19.1%, Prostate: 16.0%) compared to breast cancer (5.7%), although this difference was not statistically significant (*p* = 0.101). This apparent discrepancy warrants cautious interpretation. The decrease in mean creatinine could be influenced by factors unrelated to denosumab, such as improved hydration or recovery from prior nephrotoxic treatments within the observation period common in a real-world setting. In contrast, the elevations in creatinine can be interpreted as cases of acute kidney injury driven by other underlying conditions common in patients with advanced cancer, including dehydration, concurrent drug therapy, or the progression of the disease itself. This suggests they are an indicator of the patients’ general clinical status rather than a direct toxicity of the study drug. Also the reported “increases” might represent transient fluctuations or low-grade events meeting specific criteria without substantially impacting the overall group mean, or reflect the higher baseline creatinine observed particularly in the prostate cancer group. Given the retrospective nature, unmeasured confounders could play a role. Nonetheless, the lack of significant worsening in mean creatinine levels is reassuring and generally consistent with denosumab’s known renal safety profile [7]. Other adverse events like infections (numerically highest in prostate cancer) and malignant hypercalcemia (numerically highest in lung cancer) did not show significant intergroup differences, potentially limited by sample size.

Unsurprisingly, overall survival (OS) and post-denosumab survival were significantly different among the groups, with lung cancer patients having the shortest median survival, followed by prostate and then breast cancer (*p* < 0.005 for both). This reflects the underlying prognosis associated with the primary malignancy and its systemic control, rather than a direct survival benefit from denosumab itself in the metastatic setting [28]. Denosumab’s primary role in patients with established bone metastases is palliative—reducing skeletal morbidity and preserving quality of life [4,13]. The shorter survival observed in the lung cancer group (median PFS 6.3 months) might partially explain the higher hypocalcemia trend if these patients, due to deteriorating overall condition, had difficulty maintaining adequate oral calcium/vitamin D intake, as speculated. In terms of SRE-free survival, in Figure 1, demonstrates a sharp decline around the 50-month mark. This is attributed to the small number of patients remaining at risk at this late time point, as indicated in the accompanying ‘number at risk’ table. Given that the median follow-up period of our study was 27.5 months, the statistical power at later time points is limited. Therefore, a single skeletal-related event in a small remaining cohort results in a proportionally large drop in the SRE-free survival percentage. This observation is considered a statistical artifact rather than a reflection of a clinically significant increase in SRE risk at that specific time.

### Limitations

The retrospective design is susceptible to selection bias, missing data, and confounding variables (e.g., specific chemotherapy regimens, performance status changes, baseline vitamin D levels, precise reasons for creatinine changes). The sample size, particularly for the prostate cancer group, is relatively small, limiting the statistical power to detect smaller differences between groups. The absence of a direct comparator group (like zoledronic acid or no bone-targeting agent) restricts conclusions about relative efficacy or safety. Furthermore, the laboratory follow-up was limited to 6 months for this analysis, and systematic data on osteonecrosis of the jaw (ONJ) were not specifically presented.

## 5. Conclusions

This real-world study confirms denosumab’s efficacy in reducing SREs in patients with bone metastases from breast, lung, and prostate cancer, but highlights that hypocalcemia risk varies by tumor type. Lung cancer patients are most vulnerable, showing a significant calcium decline and the highest rate of clinical hypocalcemia, necessitating vigilant monitoring and proactive supplementation, especially in the initial 3–6 months. While breast cancer patients also require routine surveillance due to a significant calcium drop, this effect was not observed in our prostate cancer cohort, though this finding requires cautious interpretation given the sample size. Therefore, tailoring biochemical monitoring strategies based on the primary malignancy is essential to maximize the benefit–risk profile of denosumab.

## Figures and Tables

**Table 1:** Distribution of sociodemographic and clinical characteristics of the patients.

Variables		N	%
**Gender**	Male	76	52.1
	Female	70	47.9
**Age**	Mean ± std 58.3 ± 13.5		
**ECOG**	Median (min–max) 0 (0–3)		
**Comorbidities**	CKD	22	
	COPD	10	
	CAD	33	
	HT	67	
	DM	31	
**Cancer Type**	Breast	53	36.3
	Prostate	25	17.1
	Lung	68	46.6
**Treatment type**	Chemotherapy	66	45.2
	Chemotherapy + targeted therapy	17	11.6
	Chemotherapy + immunotherapy	15	10.2
	Hormonotherapy	1	0.06
	Chemotherapy + hormonotherapy	47	32.2
**SRE**	No	93	63.7
	Yes	53	36.3
**Calcium level (mg/dL)**	Before denosumab	9.3	7.9–14.1
	3rd month	9.0	6.1–11.2
	6th month	9.0	6.3–12.7
**Magnesium level (mg/dL)**	Before denosumab	2.0	1.1–2.9
	3rd month	2.0	1.1–2.3
	6th month	2.0	1.0–2.9
**Creatinine level (mg/dL)**	Before denosumab	0.78	0.39–4.48
	3rd month	0.68	0.50–5.20
	6th month	0.64	0.34–4.10
**Denosumab months**	Median 12.3 (0.2–61.8)		
**Radiotherapy**	No	57	39
	Yes	89	61
**Surgery**	No	133	91
	Yes	13	9
**Follow-up, months**	Median 27.5 (1.5–172.2)		

CAD: Coronary artery disease; CKD: Chronic kidney disease; COPD: Chronic obstructive pulmonary disease; dL: deciliter; DM: Diabetes Mellitus; ECOG: Eastern Cooperative Oncology Group; HT: Hypertension; mg: milligram; SRE: Skeletal-related event; std: standard deviation.

**Table 2:** Effect of Denosumab administration on different cancer types (breast, prostate, and lung) on frequency of skeletal-related event, toxicities, and pathological fracture.

	Breast (n/%)	Prostate (n/%)	Lung (n/%)	*p*-Value X^2^-Value
Skeletal-related event (before denosumab)	23 (43.4%)	7 (28%)	23 (33.8%)	0.354 2.079
Any toxicity	10 (18.9%)	6 (24%)	26 (38.2%)	0.056 5.787
Hypocalcemia	3 (5.6%)	1 (4%)	7 (10.2%)	0.459 * 1.703
Creatinine elevation	3 (5.7%)	4 (16%)	13 (19.1%)	0.101 4.698
Infection	6 (11.3%)	5 (20%)	12 (17.6%)	0.593 1.308
Malign hypercalcemia	10 (18.9%)	1 (4%)	13 (19.1%)	0.173 * 3.533
Skeletal-related event (after denosumab)	5 (9.4%)	4 (16%)	6 (8.8%)	0.668 * 1.236

Data were evaluated by Pearson Chi Square test or (*) Fisher’s Exact Chi Square Test (*p* < 0.05). n: number of patients.

**Table 3:** Grades of adverse events under denosumab therapy.

	Grade			
Event	1	2	3	4
Hypocalcemia, #	1	7	3	-
Infection, #	-	4	15	4
Creatinine Elevation, #	12	8	-	-

#: number of patients.

## Data Availability

The data that support the findings of this study are available on request from the corresponding author, [S.O.O]. The data are not publicly available due to ethical reasons.
